# The Tumor Vessel Targeting Strategy: A Double-Edged Sword in Tumor Metastasis

**DOI:** 10.3390/cells8121602

**Published:** 2019-12-10

**Authors:** Xiaobo Li, Yong Li, Weijin Lu, Minfeng Chen, Wencai Ye, Dongmei Zhang

**Affiliations:** 1 College of Pharmacy, Jinan University, No. 601, Huangpu Road West, Guangzhou 510632, China; 2 Guangdong Province Key Laboratory of Pharmacodynamic Constituents of Traditional Chinese Medicine and New Drugs Research, Jinan University, Guangzhou 510632, China

**Keywords:** tumor metastasis, angiogenesis, vessel targeting drugs, pro-metastasis risk

## Abstract

Tumor vessels provide essential paths for tumor cells to escape from the primary tumor and form metastatic foci in distant organs. The vessel targeting strategy has been widely used as an important clinical cancer chemotherapeutic strategy for patients with metastatic tumors. Our review introduces the contribution of angiogenesis to tumor metastasis and summarizes the application of Food and Drug Administration (FDA)-approved vessel targeting drugs for metastatic tumors. We recommend the application and mechanisms of vascular targeting drugs for inhibiting tumor metastasis and discuss the risk and corresponding countermeasures after vessel targeting treatment.

## 1. Introduction

Tumor metastasis is the main cause of cancer-induced death. More than 90% of mortality from cancer is due to metastasis [1]. Tumor vessels are necessary for tumor growth and metastasis. Irregular and tortuous tumor vessels have low pericyte coverage with high permeability, providing favorable conditions for tumor metastasis. Cancer cell dissemination via blood vessels (hematogenous spread) plays a vital role in the metastasis cascade [2], which predominantly involves local invasion, intravasation, circulation, extravasation, micrometastasis formation and metastatic colonization [3].

Based on the importance of blood vessels for tumor progression, vessel targeting strategies have been developed. The Food and Drug Administration (FDA) has approved angiogenic inhibitors, including bevacizumab, ramucirumab, sunitinib, sorafenib, regorafenib, vandetanib, cabozantinib, lenvatinib and aflibercept, for metastatic tumors. These agents block angiogenic signaling pathways and blood supply to metastatic tumor cells. The vessel targeting strategy affects the pre-metastatic niche, which suppresses angiogenesis and the inflammatory response, leading to reduced metastatic foci formation [4,5]. The vessel targeting strategy also leads to vessel normalization with decreased interstitial fluid pressure (IFP) and enhanced vessel perfusion, which inhibits disseminated cells’ intravasation [6].

However, it remains controversial in the application of vessel targeting therapy. Many investigations have shown that vessel targeting treatment leads to tumor metastasis. The disrupted endothelial cell barrier facilitates tumor cell extravasation. Hypoxia induced by vessel targeting treatment contributes to tumor cell epithelial to mesenchymal transition (EMT) and metabolism shift, directly leading to tumor cell invasion and metastasis [7,8]. In addition, hypoxia induces bone marrow derived cell (BMDC) recruitment, which indirectly facilitates tumor cell metastasis by inducing angiogenesis and producing chemokines that recruit metastatic cells to distant organs [9].

In this review, we provide an overview of angiogenesis in tumor metastasis and summarize the application of a vessel targeting strategy to metastatic cancer. We introduce the anti-metastatic effects of the vessel targeting strategy in cancer patients and the underlying mechanisms. We also analyze the risk of vessel targeting agents that induce metastasis after treatment and propose therapies to address this problem.

## 2. Angiogenesis and Metastasis

**Figure 1:**
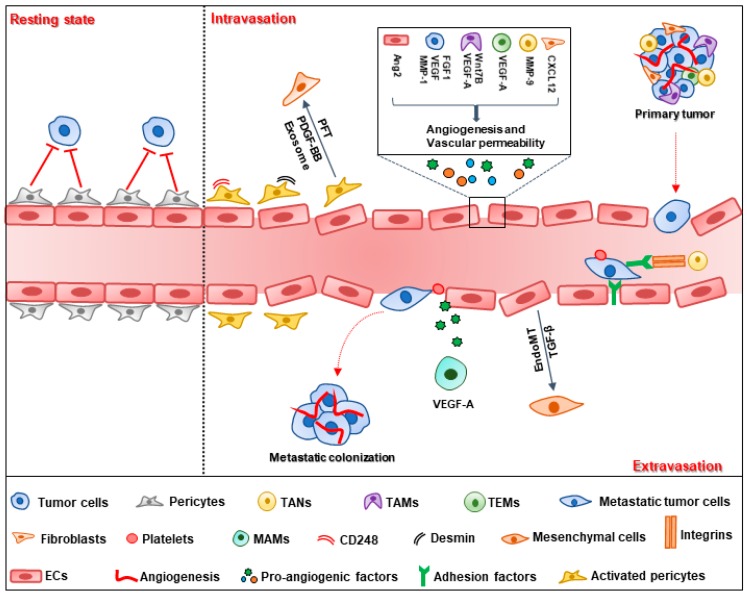
Schematic representation of the metastatic cascade. Inside the primary tumor, tumor cells produce vascular endothelial growth factor (VEGF), and fibroblast growth factor 1 (FGF1) to increase vessel density and interact with endothelial cells via the VEGF and matrix metalloproteinase-1 (MMP-1) signaling pathways to enhance vessel permeability, which provides favorable conditions for tumor metastasis. Endothelial cells (ECs) and pericytes constitute the physical barrier for tumor vessels. Pericytes, in the resting state, suppress tumor intravasation; however, certain populations of activated pericytes and cells undergone pericyte–fibroblast transition (PFT) lose their role as barriers to tumor vessels and facilitate metastasis. Many other cells in the tumor microenvironment, including tumor-associated neutrophils (TANs), tumor associated macrophages (TAMs) and Tie2-expressing macrophages (TEMs), are involved in the pro-metastatic effects, which contribute to angiogenesis and vascular permeability by secreting pro-angiogenic factors, such as VEGF-A, Wnt7B, MMP-9 and C–X–C motif chemokine ligand 12 (CXCL12). During the process of extravasation, tumor cells first adhere to ECs via various adhesion factors. Neutrophils potentiate tumor cell adhesion and transendothelial migration through the integrins/intercellular cell adhesion molecule-1 (ICAM-1) signaling pathway. Endothelial to mesenchymal transition (EndoMT), under transforming growth factor-β (TGF-β) enhances vessel permeability and contributes to the transmigration of metastatic cells. Platelets and metastasis-associated macrophages (MAMs) also enhance extravasation through opening the endothelial barrier. Finally, only a minority of disseminating cells successfully form metastatic foci and colonize in distant organs with the help of angiogenesis. The neovessels provide the necessary oxygen and nutrients for metastatic tumor growth.

Tumor vessels provide sufficient oxygen and nutrients for tumor growth and metastasis. Angiogenesis is a critical process for tumor vessel formation. In 1971, Folkman first introduced the tumor angiogenesis theory, indicating that angiogenesis is a consecutive process in which neovessels grow from preexisting vessels [10]. Compared with normal vessels, tumor vessels are arranged chaotically, with discontinuous pericyte coverage and high permeability [11]. For metastatic tumor cells, they detach from the primary tumor and pass through the tumor-blood barrier with the help of extracellular matrix proteinase, and then the cells transmigrate the endothelium and circulate in the blood flow. For survival, circulating tumor cells (CTCs) need to overcome blood flow sheer stress and escape the anti-tumor surveillance of immune cells. Finally, disseminated tumor cells extravasate and form metastatic colonization in a secondary site (Figure 1) [12,13].

### 2.1. Tumor Cell Intravasation

Tumor cell intravasation depends on various factors, including the vasculature properties and stromal cells in the tumor microenvironment [14]. Vessels more than 100 μm in diameter in the primary tumor may provide favorable conditions for tumor intravasation with respect to the liver metastasis of colorectal tumors [15]. Vessel density is the other factor that determines tumor intravasation. Fibroblast growth factor 1 (FGF1) is a pro-angiogenic factor that promotes MCF-7 breast cancer cell intravasation via increasing vessel density [16]. The blocking of vascular endothelial growth factor (VEGF) with anti-VEGF antibody was observed to decrease vessel density and suppress intravasation of human prostate carcinoma cells [17]. Tumor intravasation is closely related to the vascular barrier consisting of endothelial cells and pericytes. To enhance intravasation, malignant tumor cells release matrix metalloproteinase-1 (MMP-1) and induce high vascular permeability via the activation of protease-activated receptor-1 (PAR1) on endothelial cells. The disrupted endothelial cell barrier provides a conduit for tumor cell intravasation. Deletion of MMP-1 leads to decreased vascular permeability and reduced transendothelial migration [18]. VEGF-B secreted by tumor cells also facilitates tumor intravasation by modulating vascular structure, which increases vascular permeability and leakage, promoting tumor cell metastasis in human and mouse tumor models [19]. Except for tumor cells, endothelial cells automatically produce angiopoietin 2 (Ang2), which interacts with integrin β1 and results in reduced barrier function, leading to enhanced transendothelial migration [20]. Pericytes are another physical barrier for tumor vessels, as they attach to the endothelial tube and participate in the formation and stability of blood vessels [21]. Pericytes have dual effects on tumor intravasation. In the resting state, vessels with high pericyte coverage often display increasing stabilization and normalization compared with normal vessels, which prohibits disseminated tumor cell intravasation into the blood stream [22]. Genetic and pharmaceutical deletion of pericyte results in increased vascular leakage and intratumoral hypoxia, leading to EMT-driven metastasis [23,24]. However, pericytes can be activated under the effect of the tumor microenvironment (TME). Certain subpopulations of pericytes, such as endosialin (CD248) and desmin-overexpressing pericytes, increase tumor vessel permeability; thus, directly promoting tumor cell intravasation [25,26]. Activated pericytes indirectly promote tumor metastasis via transition into fibroblasts upon platelet derived growth factor-BB (PDGF-BB) treatment. Co-injection of pericyte–fibroblast transition (PFT) cells with less-metastatic cells facilitates tumor cell intravasation in the primary tumor [27]. PFT is also mediated by tumor-secreted exosomes through the bone morphogenetic proteins (BMPs), phosphatidylinositol 3-kinase (PI3K)/protein kinase B (AKT) and MAP kinases (MEK)/extracellular regulated protein kinase (ERK) signaling pathways [28]. Fibroblasts increase vascular permeability to promote tumor intravasation via enhancing C–X–C motif chemokine ligand 12 (CXCL12) expression. Fibroblast-derived CXCL12 is associated with poor overall survival (OS) in breast cancer patients [29].

As an important component of the TME, immune cells also play a role in tumor cell intravasation. Tie2-expressing macrophages (TEMs) are enriched in the TME with high expression of VEGF-A, which mediates loosening of vascular junctions and enhances vascular permeability, promoting tumor cell intravasation [30]. Tumor-associated macrophages (TAMs) perform pro-angiogenic effects through their response to colony-stimulating factor-1 (CSF-1) and Ang2 [31,32]; meanwhile, TAMs produce VEGF-A and Wnt7B [33,34]. These effects mediated by TAMs, facilitate tumor cell intravasation by increasing the density of blood vessels [35]. Tumor-associated neutrophils (TANs) contribute to tumor intravasation by inducing neovessel formation via MMP-9. Inhibition of neutrophil infiltration by interleukin 8 (IL-8) neutralization leads to diminished tumor angiogenesis and intravasation [36]. These data strongly support a role of stromal cells in the tumor environment in the promotion of intravasation via vessel formation and stabilization [37].

### 2.2. Tumor Cell Extravasation

Tumor cells adhere to endothelial cells and then transmigrate into the extravascular stroma through paracellular migration, in which tumor cells migrate between two endothelial cells via cellular rearrangements and disruption of inter-endothelial cell junctions [38]. Multiple factors are involved in the adhesion stage of extravasation, including selectins, integrins, N-cadherin, CD44, MUC1, and intercellular cell adhesion molecule-1 (ICAM-1) [39]. Dynamic imaging shows that integrin β1 contributes to tumor protrusion formation and mediates adhesion to the subendothelial matrix, which protects tumor cells from detachment into the blood flow and facilitates tumor extravasation [40]. In addition, ICAM-1 participates in tumor cell adhesion and transendothelial migration [41]. ICAM-1 is expressed both in endothelial and tumor cells. The binding of ICAM-1 with αLβ2 and β2 integrin on neutrophils facilitates tumor cell adhesion and extravasation [42,43]. During the process of extravasation, tumor cells and other cells, including endothelial cells, platelets and myeloid cells co-contribute to cell extravasation. The endothelial to mesenchymal transition (EndoMT) is a new type of transdifferentiation in which endothelial cells acquire mesenchymal or myofibroblastic markers, such as α-smooth muscle actin (α-SMA) and type I collagen, with the stimulation of TGF-β [44]. EndoMT leads to endothelial cytoskeleton remodeling and increased vessel permeability, which facilitates melanoma extravasation [45]. Platelets promote tumor extravasation by inducing an invasive, mesenchyme-like phenotype in tumor cells by activating the TGF-β/Smad/nuclear factor-κappa B (NF-κB) pathway. Platelets interact with endothelial cells through the ATP/P2Y_2_ signaling pathway, which facilitates tumor cell extravasation by opening the endothelial barrier [46]. Myeloid progenitor cells are also involved in tumor extravasation by transdifferentiating into metastasis-associated macrophages (MAMs) and secreting VEGF-A to increase vascular permeability; thus, enhancing the extravasation of cancer cells [35]. However, only a minority of disseminated tumor cells form metastasis in the secondary site because most tumor cells are cleared away by immune cells or apoptosis after extravasation. To form a metastatic tumor, tumor cells need to proliferate and colonize in the target organ with the help of neovessels, which provide nutrients and oxygen for secondary tumor growth [1].

## 3. Application of Vessel Targeting Drugs in Metastatic Cancer

The FDA has approved a series of anti-angiogenic drugs applied for chemotherapy of malignant tumors. Anti-angiogenic drugs are mainly classified into three types: monoclonal antibodies, tyrosine kinase inhibitors (TKIs) and fusion peptides [47]. These anti-angiogenic drugs are widely applied in a range of malignant metastatic cancers, such as colorectal cancer, non-small cell lung cancer, renal cell cancer and medullary thyroid cancer (Table 1).

### 3.1. Monoclonal Antibody Therapy

A monoclonal antibody is designed to specifically bind with a signaling molecule in angiogenesis and neutralize its function. At present, two kinds of monoclonal antibodies are approved: bevacizumab and ramucirumab.

Bevacizumab is a humanized monoclonal anti-VEGF-A antibody that was approved by the FDA as the first anti-angiogenic agent in 2004 [85]. Bevacizumab is widely applied to the treatment of metastatic colorectal cancer (mCRC), metastatic non-small cell lung cancer (mNSCLC), metastatic renal cell carcinoma (mRCC) and metastatic cervical cancer as the first and second-line agent in combination with standard chemotherapy. For instance, bevacizumab is approved for first-line treatment of mCRC in conjunction with 5-fluorouracil-irinotecan or 5-fluorouracil-oxaliplatin-based chemotherapy [48]. A large amount of clinical experimental data suggest that intravenous injection of bevacizumab prolongs the progression-free survival (PFS) and OS of patients with mCRC [49,50,51]. Bevacizumab prevents brain metastases’ formation of nonsquamous non–small cell lung cancer (nsNSCLC) in mouse models, leading to a survival benefit, which suggests that bevacizumab might benefit patients with stage III nsNSCLC who are at a high risk of developing brain metastases [52]. The combination of bevacizumab with interferon-α (IFN-α) is more effective in patients with mRCC than IFN-α treatment alone, which leads to significant improvement in PFS [53]. Bevacizumab is also effective in the treatment of metastatic cervical cancer in combination with chemotherapy, such as cisplatin and paclitaxel, which increases the OS of patients [54]. Although bevacizumab was removed by the FDA for the treatment of metastatic breast cancer, citing safety concerns, the clinical data show that bevacizumab treatment significantly prolongs PFS of metastatic breast cancer patients with an acceptable toxicity profile and is still advised for application in the clinic [55,56].

Ramucirumab is a humanized monoclonal antibody that selectively targets vascular endothelial growth factor receptor-2 (VEGFR-2) and blocks the interaction of VEGFR-2 with VEGF. Ramucirumab has been approved for application in colorectal cancer, non-small cell lung cancer and stomach adenocarcinoma or an gastroesophageal junction adenocarcinoma that has metastasized [57,58,59]. Clinical experiments have shown that the combination of ramucirumab and docetaxel prolonged PFS in patients with locally advanced or metastatic urothelial carcinoma, which further guarantees investigation in a phase III trial [60].

### 3.2. Tyrosine Kinase Inhibitor Therapy

TKIs are the second group of anti-angiogenic agents that block the activation signal of the intracellular domain after binding of the cell surface receptors and ligands. TKIs may exclusively target one receptor during angiogenesis; however, in most cases, TKIs are multitarget agents; targets include VEGFR, platelet derived growth factor receptor (PDGFR), fibroblast growth factor receptor (FGFR) and c-Kit. Sunitinib and sorafenib are the most common TKIs in the clinic [86].

Sunitinib targets include VEGFR1-3, PDGFR-α, PDGFR-β, c-Kit, colony-stimulating factor-1 receptor (CSF-1R) and RET. Sunitinib is approved by the FDA for the treatment of metastatic pancreatic cancer. Sunitinib was observed to markedly improve the two year OS of patients with metastatic pancreatic adenocarcinoma [61,62]. Sunitinib is also effective against mRCC and is applied as a first-line treatment of mRCC, since it shows superiority compared with IFN-α in a phase III trial [63,64].

Sorafenib inhibits the tyrosine phosphorylation of VEGFR-1, 2 and 3; PDGFR-β; Flt-3; and c-Kit [87]. Sorafenib is the priority drug used for advanced hepatocellular carcinoma (HCC) [65], as it inhibits the lung metastasis of HCC mediated by CD90^+^ cancer stem cells (CSCs) [66]. The combination of sorafenib and carfilzomib synergistically suppresses the metastasis of HCC via inducing tumor cell apoptosis [67]. Sorafenib may benefit patients with metastatically differentiated thyroid cancers that are resistant to radioactive iodine [68].

Regorafenib is a multikinase inhibitor that potentially targets angiogenesis-associated kinase and the mutant oncogenic kinases, including VEGFR-1 and 3, PDGFR-β, FGFR1, KIT, RET and B-RAF [69]. Regorafenib was approved in 2012 for the treatment of patients with metastatic colorectal cancer previously treated with other therapy. Later, in 2013, regorafenib was applied to patients with locally advanced, unresectable or metastatic gastrointestinal stromal tumors (GISTs) who had been previously treated with imatinib and sunitinib as third-line treatment [70]. A phase II study reported that regorafenib benefits patients with progressive metastatic osteosarcoma and other bone sarcomas by delaying disease progression and extending the PFS of patients with metastatic osteosarcomas [71].

Vandetanib selectively inhibits VEGFR-2 and VEGFR-3, blocking VEGF-stimulated endothelial cell proliferation and migration to inhibit angiogenesis. Vandetanib was approved by the FDA to treat metastatic medullary thyroid cancer (MTC) in 2011 and in 2012 by the European Medicines Agency (EMA), which was observed to improve PFS (30.5 versus 19.3 months in the placebo group) in patients with MTC [72].

Cabozantinib was also approved by the FDA to treat progressive metastatic MTC in 2012, which is a TKI against MET, RET, AXL, VEGFR-2, FLT3 and c-Kit [73]. Cabozantinib has therapeutic effects on metastatic castration-resistant prostate cancer (mCRPC) by eliminating the bone metastasis of mCRPC with pain relief [74,75]. Cabozantinib was approved as a first and second-line treatment for advanced/metastatic renal cell carcinoma based on two clinical trials [76].

Lenvatinib is a multitargeted tyrosine kinase inhibitor targeting the families of VEGFR, FGFR, PDGFRα, KIT and RET [88]. Lenvatinib was approved by the FDA to treat patients with metastatic, progressive and radioactive iodine–refractory differentiated thyroid cancer in 2015, which, thus, provides a new oral option for differentiated thyroid cancer [77]. Later, in 2018, lenvatinib was approved for the treatment of metastatic HCC patients, which had a favorable clinical activity and tolerable toxicity [78,79]. Clinical data show that lenvatinib alone improves the PFS of patients with mRCC who have disease progression after VEGF-targeted therapy [80].

### 3.3. Fusion Protein Therapy

Ziv-aflibercept is the representative agent of the third type of angiogenesis inhibitors: fusion protein. Ziv-aflibercept is composed of the extracellular domain of both VEGFR-1 and VEGFR-2 fused to the Fc region of IgG1, which interacts with VEGF-B and placental growth factor (PlGF) and halts the pro-angiogenic effects of the VEGF/VEGFR signaling pathway [81,82]. In 2012, the U.S. FDA approved aflibercept in combination with 5-fluorouracil, leucovorin and irinotecan (FOLFIRI) for the treatment of patients with mCRC that is resistant to or has progressed after oxaliplatin chemotherapy, significantly improving the OS, PFS and partial responses in patients with mCRC [83,84].

## 4. Mechanism of the Anti-Metastatic Effects Mediated by Vessel Targeting Therapies

**Figure 2:**
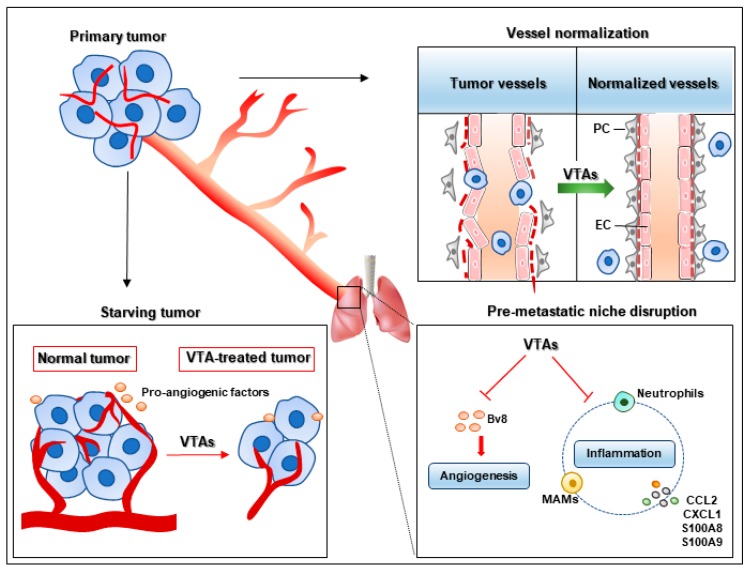
Anti-metastatic mechanisms of vessel targeting agents. Vessel targeting agents (VTAs) inhibit tumor metastasis through diverse mechanisms. First, VTAs starve tumor cells and block metastatic conduits by suppressing neovessel formation and pro-angiogenic factor production. Second, vessel targeting agents inhibit the bombina variegate 8 (Bv8)-induced vessel sprouting and the inflammation mediated by neutrophils, metastasis-associated macrophages (MAMs), C–C motif chemokine ligand 2 (CCL2) and C–X–C motif chemokine ligand 1(CXCL1) in the pre-metastatic niche; thus, disturbing pre-metastatic niche formation and inhibiting metastasis. Thirdly, vessel targeting agents lead to reduced metastasis through vessel normalization with increased endothelial cell (EC) junction and pericyte (PC) coverage and decreased vessel permeability.

Vessel-targeting therapies inhibit tumor metastasis through multiple mechanisms (Figure 2). The relevant mechanism is that cancer cells are starved to death without the supply of vessels [89]. On the one hand, angiogenesis inhibitors suppress neovessel formation with decreased vascular density and functional aberrations. The tumor cells lose their pathway to disseminate to other organs. On the other hand, these agents block the pro-angiogenic signaling pathways such as VEGF, PDGF, FGF, Ang, hematopoietic growth factor (HGF), IL-6 and so on, which regulate vessel permeability, remodeling, endothelial cell survival, proliferation and migration, leading to abnormal angiogenesis and tumor metastasis [90,91]. Here, we discuss some anti-metastatic mechanisms of vessel targeting agents that have recently aroused concern.

### 4.1. Pre-Metastatic Niche Disruption

The pre-metastatic niche provides a favorable environment for seeding and colonization of disseminated tumor cells in the second organ, which is essential for metastatic foci formation. Angiogenesis and the inflammatory response are the main characteristics of the pre-metastatic niche, which provide the necessary conditions for disseminated tumor cells to form metastatic foci and achieve colonization [92]. Regarding the role of angiogenesis in the pre-metastatic niche, anti-angiogenic agents can suppress metastasis via directly blocking metastatic routes through inhibiting neovessel sprouting. Ang2 is a potential target for anti-angiogenic agents. Anti-Ang2 antibody inhibits vessel sprouting and formation mediated by the angiogenic cytokine bombina variegate 8 (Bv8), which is produced by myeloid cells in metastatic nodules [5]. Vessel targeting agents also suppress metastasis via inflammatory responses, which affect the pre-metastatic niche through various inflammatory cells and chemokines. Ang2 neutralization inhibits the recruitment of CCR2^+^Tie2^−^ MAMs via blocking C–C motif chemokine ligand 2 (CCL2) and ICAM-1, leading to reduced metastases [5]. The neutrophil-mediated inflammatory response also facilitates tumor metastasis, as neutrophils are recruited to the pre-metastatic niche and produce leukotrienes that contribute to the colonization and metastasis of breast cancer cells in the lung [93]. TSU-68 is an antiangiogenic agent targeting multiple tyrosine kinase receptors, such as VEGFR-2, PDGFR and FGFR [94]. TSU-68 significantly inhibits the expression of inflammatory chemokines, including CXCL1, S100A8 and S100A9, which suppresses the CXCL1/C–X–C motif chemokine receptor 2 (CXCR2)-mediated inflammatory response with decreased CXCR2^+^neutrophils infiltration in the pre-metastatic niche, leading to a marked inhibition of liver metastasis [4].

### 4.2. Vessel Normalization

Vessel normalization changes tortuous and leaky vessels into straight and regular vessels, which decreases vessel permeability and IFP. The high perfusion and low IFP halt the intravasation of tumor cells, leading to decreased metastasis [6,95]. Tie1 is expressed in activated endothelial cells, which is a key regulator of angiogenesis and vascular remodeling [96]. Tie1 deficiency leads to reduced angiogenesis and increased mural cell coverage with improved vessel perfusion via activating the Ang1/Tie2 signaling pathway. The normalized tumor vessels inhibit tumor intravasation, extravasation and metastatic foci formation [97]. Directly activating Tie2 with the Ang2-binding and TIE2-activating antibody (ABTAA) complex induces tumor normalization with increased pericyte and collagen type IV basement membrane coverage, which suppresses tumor cell metastasis and extravasation in glioma, Lewis lung carcinoma (LLC) and breast cancer [98]. Vessel normalization is also associated with the hyperglycolytic metabolism of tumor endothelial cell (TEC). The genetic inhibition of the glycolytic activator PFKFB3 improves vessel perfusion and tightens the endothelial cell barrier. The normalized tumor vessels inhibit cancer cell intravasation and metastasis [99]. A number of investigations showed that pharmacologic intervention with vessel targeting drugs induces vessel normalization [100]. Bevacizumab used at a certain dose leads to vessel normalization, which is accompanied by increased pericyte coverage and vessel perfusion [101,102]. The preclinical data reveal that bevacizumab-mediated vessel normalization improves the oxygen supply in tumors and facilitates the delivery of chemotherapy drugs, which benefit patients with mCRC and metastatic breast cancer [103]. Infigratinib is an FGFR kinase inhibitor that suppresses basic FGF (bFGF)-stimulated angiogenesis and mediates vessel normalization with increased perfusion. Infigratinib treatment inhibits tumor hypoxia via vessel normalization, which significantly reduces lung metastasis in HCC models [104]. From the above studies, vessel normalization mediated by antiangiogenic agents is a direct and effective method to recover the normal functions of vessels and then suppress metastasis.

## 5. Metastasis Risk after Vessel Targeting Therapies

**Figure 3:**
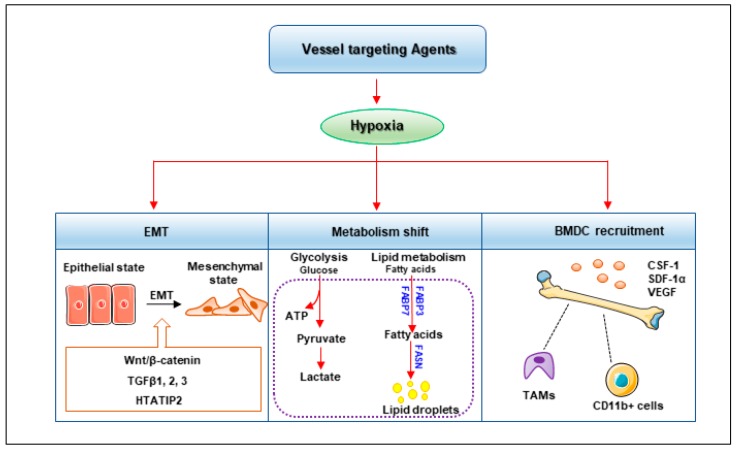
Hypoxia mediates pro-metastatic risk of vessel targeting agents. Hypoxia mediated by vessel targeting agents facilitates tumor epithelial mesenchymal transition (EMT) with decreased expression of epithelial cell markers and enhanced mesenchymal cell markers via the Wnt/β-catenin, transforming growth factor β (TGFβ) and HIV-1 Tat interactive protein 2 (HTATIP2) signaling pathways. Hypoxia contributes to tumor metabolism shift with increased glycolytic metabolism and lipid metabolism by upregulating fatty acid binding protein (FABP3), FABP7 and fatty acid synthase (FASN). Hypoxia also promotes the infiltration of bone marrow derived cells (BMDCs), including tumor associated macrophages (TAMs) and CD11b^+^ cells through colony-stimulating factor-1 (CSF-1), stromal-derived factor-1α (SDF-1α) and VEGF.

Vessel targeting therapy is not always effective for tumor metastasis [105]. Vessel targeting agents lead to vessel destabilization, including endothelial barrier disruption, which facilitates tumor cell extravasation. Sunitinib disrupts endothelial barrier integrity by decreasing the expression of vascular endothelial cadherin (VE-cadherin) and ZO-1, which maintains the endothelial cell junction. The leaky vessel post-sunitinib treatment contributes to tumor cell extravasation, which increases the number and area of metastatic lung nodules [106]. The poor vasculature with decreased vessel density and stability after vessel targeting treatment often leads to tumor hypoxia compared with neighboring tissues. Hypoxia is associated with poor patient prognosis and has been validated to promote tumor cell invasion and metastasis [107]. Anti-angiogenic agents, including DC101 (antibody against VEGFR-2) and sunitinib, promote local invasiveness and increase distant metastasis in the mouse models of pancreatic neuroendocrine cancer and glioblastoma multiforme. Mice treated with DC101 or sunitinib show severe hypoxia, especially in the micrometastases of distant organs, indicating the crucial role of hypoxia in promoting metastasis after anti-angiogenic therapy [108]. Hypoxia is the most influential factor of hypoxia-inducible factors’ (HIF) activation. The HIF family (HIF-1α, HIF-2α, HIF-3α, HIF-1β and HIF-2β) is widely expressed in solid tumors, including those of non-small cell lung cancer, breast cancer, colon cancer, head and neck cancer, gastric cancer and prostate cancer. Hypoxia/HIF induce metastasis through a series of pathways, including regulation of EMT, metabolism shift and BMDC recruitment (Figure 3) [7,9].

### 5.1. EMT

EMT is characterized by changes in cell morphology and cell-to-cell and cell-to-matrix adhesions, which endow tumor cells with migratory and invasive capacities. The phenotypic changes in EMT cells include the down-regulation of epithelial cell markers, such as E-cadherin, and high expression of mesenchymal cell markers, including N-cadherin, vimentin and metalloproteases [109,110]. HIF directly regulates EMT transcription factors, including zinc finger E-box binding homeobox (ZEB), snail, slug and twist, which have prognostic significance in tumor metastasis [111]. The HIF pathway also indirectly promotes EMT through a number of cell signaling pathways, including Notch, TGF-β, Sonic Hedgehog and Wnt, which all play key roles in the process of EMT [112,113]. Bevacizumab was reported to induce EMT via activation of Wnt/β catenin signaling in glioblastoma progression, which is accompanied by the increased expression of mesenchymal markers, such as N-cadherin and vimentin [114,115]. Bevacizumab also induces the upregulation of EMT-induced factors including TGFβ1, 2 and 3 in breast cancer patients, which is mediated by acute hypoxia after bevacizumab treatment. Sorafenib treatment also promotes invasiveness and the metastasis of orthotopic HCC tumors in mice by EMT. Sorafenib downregulates the expression of HIV-1 Tat interactive protein 2 (HTATIP2) via the Janus kinase (JAK)-signal transducer and activator of transcription 3 (STAT3) signaling pathway, leading to induced EMT in HCC cells [116].

### 5.2. Metabolism Shift

A study has demonstrated that metabolism reprogramming induced by anti-angiogenesis drugs facilitates tumor metastasis. Bevacizumab treatment leads to a significant upregulation of glycolytic genes, which results in a metabolism shift towards anaerobic glycolysis with enhanced lactate production, promoting cancer cell invasion and metastasis in glioblastoma [117,118]. Lipid metabolism also plays a crucial role in metastasis mediated by angiogenic inhibitors. Bevacizumab treatment leads to lipid droplet (LD) accumulation by promoting the expression of fatty acid binding protein 3 (FABP3) and FABP7 [119]. Sunitinib and sorafenib both show clinical benefits to cancer patients. However, they are reported to induce tumor regrowth and metastasis after drug withdrawal. Cancer cell metabolism is altered from glycolytic metabolism to lipid metabolism after the withdrawal of angiogenesis inhibitors. Citric acid cycle activity increases accompanied by reduced glycolysis after treatment ends. Fatty acid synthase (FASN) is a critical enzyme in lipogenesis [120], and blocking FASN inhibits tumor regrowth and metastasis after the withdrawal of sunitinib treatment. The result has been validated in a colorectal cancer xenograft and transgenic mouse model of mammary carcinoma [121,122].

### 5.3. BMDC Recruitment

Several investigations show that vessel targeting agents enhance tumor metastasis by recruiting bone marrow-derived pro-angiogenic cells. The infiltrated BMDCs promote tumor revascularization and metastasis. Hypoxia induced by vessel targeting agents upregulates the expression of stromal-derived factor-1α (SDF-1α), which is a chemoattractant of CD45^+^myeloid cells and induces recruitment of Tie2, VEGFR-1, CD11b and F4/80-expressing subtypes of myeloid cells; myeloid cells then promote angiogenesis and induce tumor cell intravasation, dissemination and metastasis [123,124]. CD11b^+^myeloid cells contribute to hepatic metastasis through downregulation of angiopoietin-like 7 (ANGPTL7). Depletion of CD11b^+^myeloid cells or overexpression of ANGPTL7 significantly inhibits hepatic metastasis formation and angiogenesis [125]. Immune-suppressive cells, such as TAMs, participate in tumor angiogenesis and metastasis, inducing tumor cell invasion, extravasation and survival in the metastatic niche. The recruitment and polarization of TAMs are driven by a series of growth factors, including CSF-1, granulocyte-macrophage colony-stimulating factor (GM-CSF) and CCL2, which are secreted by tumor cells and TAMs, and then produce epidermal growth factor (EGF), MMP-9, MMP-2 and urokinase plasminogen activator (uPA) to promote tumor cell metastasis [126,127]. TAMs play a central role in bevacizumab treatment. High TAM infiltration and variations in genes regulating TAM-related functions such as *TBK1*, *CCL2*, *CCL18* and *IRF3* predict poor outcome in metastatic patients treated with bevacizumab [128,129]. Sorafenib treatment enhances the infiltration of F4/80 and CD11b-positive cells in the peripheral blood of HCC xenograft model via CSF-1, SDF-1α and VEGF, which are key cytokines for macrophage recruitment. The combination of sorafenib with macrophage-targeting drugs including zoledronic acid (ZA) and clodrolip suppresses the recruitment of macrophage and further reduces lung metastasis [130].

## 6. Discussion

Hematogenous metastasis is the principal pathway for malignant tumor metastasis. Vessel targeting treatment can inhibit metastasis through starving tumor cells, inducing vessel normalization and disrupting the pre-metastatic niche. However, vessel targeting treatment still poses a pro-metastatic risk for patients. Here, we mainly discuss some potential methods to circumvent the problem.

Hypoxia is considered to be the greatest hindrance to vessel targeting treatment. Therefore, a combination medication of a vessel targeting treatment with a hypoxia targeting therapy is a better choice in the clinic. To monitor hypoxia, dynamic contrast-enhanced magnetic resonance imaging (DCE-MRI) and 18F-Fluoromisonidazole (18F-FMISO) are the most effective methods for tumor areas. In addition, multiple HIF inhibitors have been investigated and demonstrated to block the hypoxia pathway and exert antitumor effects [131,132]. These inhibitors suppress the mRNA expression, protein synthesis, protein degradation and dimerization, DNA binding and transcriptional activity of HIF-1 and HIF-2, and some of inhibitors have progressed into clinical trials [133]. Hypoxia-directed gene therapy is another strategy achieved by designing therapeutic genes that are controlled by hypoxia response elements (HREs) or other promoters under HIF-1 activation. A therapeutic gene was used to selectively activate prodrug and increase drug cytotoxicity under hypoxia conditions [134,135]. Bioreductive prodrugs target tumor hypoxia in an oxygen-sensitive manner, which are activated by endogenous oxidoreductases and metabolized to cytotoxins, including nitro compounds, N-oxides, quinones and metal complexes [136].

Both hypoxia and abnormal tumor vasculature induce dysfunction of a tumor’s immune microenvironment, which regulates the functions of the innate and adaptive immune system towards immunosuppression [137,138,139,140]. The expression of programmed cell death 1 ligand 1 (PD-L1) on dendritic cells (DCs), TAMs and tumor ECs is also increased [141,142]. Anti-angiogenic agents normalize abnormal vessels, which facilitate T cell recruitment and decrease the infiltration of pro-tumor immune cells, including regulatory T cells, M2-like TAMs and myeloid-derived suppressor cells (MDSCs) [143,144,145]. Therefore, a potential strategy is to combine anti-angiogenesis agents with immunotherapy, especially T-cell based immunotherapy. Inhibition of VEGFA and Ang-2 normalizes tumor vessels and increases IFNγ^+^ CD8^+^ T cells’ extravasation and accumulation, which further enhances the antitumor effects of PD-1 inhibitors [146,147]. Moreover, the combination of VEGFR-2 and PD-L1 antibodies induces high endothelial venules (HEVs) to facilitate IFNγ^+^ CD4^+^ and IFNγ^+^ CD8^+^ lymphocyte infiltration in breast cancer and pancreatic neuroendocrine tumors, finally leading to tumor cell apoptosis and necrosis [148]. This combination therapy has achieved certain results in the treatment of metastatic cancer. The combination of anti-angiogenic agents with PD-1/PD-L1 inhibitors is safe and tolerable in patients with metastatic, clear cell, renal cell carcinoma [149] and metastatic mucosal melanoma [150]. The combined application of atezolizumab (anti-PD-L1) with bevacizumab, carboplatin and paclitaxel significantly prolongs PFS and OS in patients with metastatic nsNSCLC [151]. These data indicate that the combination of anti-angiogenic therapy with immunotherapy can synergistically benefit patients with metastatic cancer.

Drug resistance is also associated with the failure of anti-angiogenic therapies in clinical applications. Vessel cooption is a key mechanism mediating resistance to anti-angiogenic therapy, in which tumor cells hijack the pre-existing vasculature to support tumor growth without the need for angiogenesis [152]. Vessel cooption is commonly found in human lung, liver and brain metastases [153]. The co-opted vessels facilitate metastatic foci formation and colonization, leading to the failure of treatment with bevacizumab, sunitinib and ZD6474 [154,155,156]. Therefore, combined inhibition of angiogenesis and vessel cooption might be an optimized strategy for the application of vessel targeting drugs in the metastatic tumors.

## 7. Conclusions

Angiogenesis provides advantageous conditions for tumor metastasis, providing an avenue for the development of antiangiogenic drugs. The vessel targeting strategy is an important strategy for metastatic cancer patients in the clinic, though it creates a risk for tumor metastasis under certain conditions. Strategies for monitoring and decreasing the pro-metastatic risk of vessel targeting agents should be further developed.

## Figures and Tables

**Table 1:** Food and Drug Administration (FDA)-approved vessel targeting drugs for metastatic cancer.

Agent	Type of Inhibitor	Targets	Clinical Application
Bevacizumab	Monoclonal antibody	VEGF-A	Metastatic colorectal cancer [48,49,50,51] Metastatic nonsquamous non-small cell lung cancer [52] Metastatic renal cell carcinoma [53] Metastatic cervical cancer [54] metastatic breast cancer [55,56]
Ramucirumab	Monoclonal antibody	VEGFR-2	Metastatic colorectal cancer [57] Metastatic non-small cell lung cancer [58] Metastatic stomach adenocarcinoma [59] Metastatic gastroesophageal junction adenocarcinoma [59] Metastatic urothelial carcinoma [60]
Sunitinib	Tyrosine kinase inhibitor	VEGFR-1, VEGFR -2, VEGFR -3, PDGFR-α, PDGFR-β, c-Kit, CSF-1R, RET	Metastatic pancreatic cancer [61,62] Metastatic renal cell carcinoma [63,64].
Sorafenib	Tyrosine kinase inhibitor	VEGFR-1, VEGFR -2, VEGFR -3, PDGFR-β, Flt-3, c-Kit	Metastatic hepatocellular carcinoma [65,66,67] Metastatic thyroid cancer [68]
Regorafenib	Tyrosine kinase inhibitor	VEGFR-1, VEGFR -3, PDGFR-β, FGFR-1 KIT, RET and B-RAF	Metastatic colorectal cancer [69] Metastatic gastrointestinal stromal tumor [70] Metastatic osteosarcoma [71]
Vandetanib	Tyrosine kinase inhibitor	VEGFR-2, VEGFR-3, EGFR, RET	Metastatic medullary thyroid cancer [72]
Cabozantinib	Tyrosine kinase inhibitor	MET, RET, AXL, VEGFR-2, FLT3, c-Kit	Metastatic medullary thyroid cancer [73] Metastatic castration-resistant prostate cancer [74,75] Metastatic renal cell carcinoma [76]
Lenvatinib	Tyrosine kinase inhibitor	VEGFR-1, VEGFR-2, VEGFR-3 FGFR, PDGFRα, KIT and RET	Metastatic differentiated thyroid cancer [77] Metastatic hepatocellular carcinoma [78,79] Metastatic renal cell carcinoma [80]
Aflibercept	Fusion protein	VEGF-A, VEGF-B, PlGF	Metastatic colorectal cancer [81,82,83,84]
